# Case Report: Delayed gastrointestinal structural complications requiring surgery following massive paint thinner ingestion in an adult

**DOI:** 10.3389/ftox.2026.1719604

**Published:** 2026-02-26

**Authors:** Hayeon Lee, Haewon Lee, Min Chang Kang, Eunhae Um, Hyunwoo Sun

**Affiliations:** 1 Department of Critical Care Medicine, Uijeongbu Eulji Medical Center, Eulji University College of Medicine, Uijeoungbu, Republic of Korea; 2 Department of Surgery, Ilsan Paik Hospital, Inje University College of Medicine, Goyang-si, Republic of Korea

**Keywords:** drug intoxication, gastrointestinal stricture, hydrocarbon toxicity, paint thinner ingestion, surgical management

## Abstract

**Background:**

Paint thinners, which contain volatile aromatic hydrocarbons like toluene and xylene, are primarily associated with acute systemic toxicity (e.g., central nervous system depression, metabolic acidosis) following inhalation. Case reports detailing severe, delayed gastrointestinal (GI) structural complications in adults after massive oral ingestion, which necessitate extensive surgical intervention, remain exceedingly rare in the medical literature.

**Case description:**

A 68-year-old male with a history of hypertension and chronic alcoholism presented 8 h after mistakenly ingesting approximately 500 mL of paint thinner while intoxicated. On admission, the patient exhibited stupor, hypotension (74/49 mmHg), bradycardia (75 bpm), hypothermia (34.0 °C), and significant hematochezia. Though systemic symptoms stabilized after initial intensive care, he developed recurrent, refractory episodes of ileus over the following 3 weeks, despite conservative management. Abdominal computed tomography (CT) scans revealed multisegmental wall thickening and lumen stricture in the distal jejunum and ileum, suggestive of enteritis or inflammatory bowel disease. Surgical exploration on Hospital Day (HD) 27 confirmed the progression to irreversible structural damage, revealing severe adhesion, a continuous global stricture in the terminal ileum (extending approximately 80 cm from the ileocecal valve), a skip lesion stricture proximally, and a suspected ileocecal fistula. An ileocecectomy was performed after resection of a 132 cm segment of small bowel. Pathology confirmed chronic, transmural inflammation and ulceration.

**Conclusion:**

This case demonstrates that massive oral paint thinner ingestion can bypass typical protective mechanisms, leading to severe, delayed, and localized structural GI failure, particularly in the terminal ileum. In patients who present with progressive mechanical sequelae (refractory ileus and hematochezia) following massive hydrocarbon ingestion, a high index of suspicion for developing chronic structural lesions is warranted, suggesting that timely surgical intervention may be critical to expedite patient recovery.

## Introduction

Paint thinners are complex mixtures of volatile organic solvents, predominantly comprising aromatic hydrocarbons such as toluene, xylene, and N-hexane. The toxicological profile of these agents is well-documented, primarily relating to acute neurotoxicity, myotoxicity, hepatotoxicity, and cardiotoxicity, generally following exposure via inhalation or recreational abuse (Mickiewicz and Gomez, 2001; Tormoehlen et al., 2014). Existing case reports largely focus on these systemic effects or on accidental ingestion by pediatric patients, often resulting in aspiration pneumonitis (Beamon et al., 1976; Truemper et al., 1987; Malingre et al., 2002; Zaidi et al., 2007; Agin et al., 2016).

However, the consequences of massive oral ingestion of paint thinners by adults, particularly those requiring surgical management for severe gastrointestinal (GI) complications, are rarely reported (Argo et al., 2010; Rahimi et al., 2015; Dhibar et al., 2018). This route of exposure presents a distinct challenge, as hydrocarbons are lipophilic and exert direct cellular damage upon contact with the GI mucosa. We present a unique case of a 68-year-old male who ingested an estimated 500 mL of paint thinner, leading to severe, delayed, and localized transmural necrosis resulting in extensive small bowel stricture and fistula formation, necessitating a major ileocecectomy. This report contributes to the scarce literature by highlighting the potential for delayed structural failure and the critical role of timely surgical intervention in managing this rare complication.

## Case presentation

A 68-year-old male with a past medical history of hypertension and chronic alcoholism was transferred to our institution on 3 May 2023, due to mental change and severe hematochezia. The patient reported that, after consuming approximately three bottles of *Makgeolli* (Korean rice wine), he mistakenly consumed paint thinner that was stored in an identical liquor bottle, ingesting an estimated 500 mL. He did not vomit after ingestion and was found by his son approximately 8 h later in a stuporous state.

On initial presentation, the patient was drowsy (mental stupor) and had vital signs indicating acute toxicity and instability: blood pressure 74/49 mmHg, heart rate 75 bpm, respiratory rate 15/min, oxygen saturation 95% (with 3L nasal cannula), and hypothermia (34.0 °C). Initial laboratory analysis confirmed severe metabolic derangement, including severe metabolic acidosis (Arterial Blood Gas pH 7.23, Lactate 7.1 mMol/L) and acute kidney injury (BUN/Creatinine 17.9 mg/dL/1.63 mg/dL). Other key values included White Blood Cell count 9.0*10^3^/ul, Hemoglobin 16.5 g/dL, Potassium 3.8 mMol/L, CK-MB 23.0 ng/mL, and Troponin-I 0.024 ng/mL. Initial management included the administration of norepinephrine for low blood pressure and sodium bicarbonate boluses and continuous infusion for suspected metabolic acidosis. He was admitted to the Surgical Intensive Care Unit (SICU).

### Hospital course

The patient exhibited persistent hematochezia and passing stools with a noticeable thinner odor for the first 3 days, indicating direct GI mucosal damage. By Hospital Day (HD) 3, systemic symptoms stabilized, norepinephrine was tapered off, and the patient was transferred to the general ward.

However, the GI course was complicated. On HD 4, he developed symptoms of ileus, necessitating the insertion of an L-tube, which drained 700 mL. Over the subsequent weeks, the ileus symptoms waxed and waned, with the L-tube being removed and reinserted multiple times (HD 10, HD 14, HD 18, HD 24). On HD 24, the L-tube drained 800 mL, confirming severe obstruction. Throughout this period, the patient developed intermittent fever and was treated with broad-spectrum antibiotics (Piperacillin/Tazobactam).

### Imaging and pathology

**FIGURE 1:**
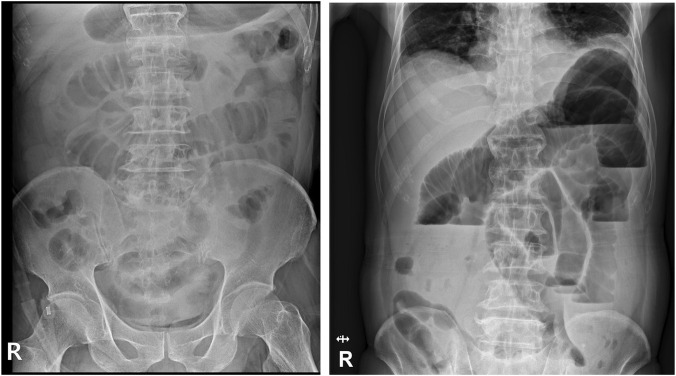
Abdominal radiographs obtained on hospital day (HD) 1 and HD 13 showing progressive small bowel dilatation with multiple air–fluid levels, consistent with worsening ileus.

**FIGURE 2:**
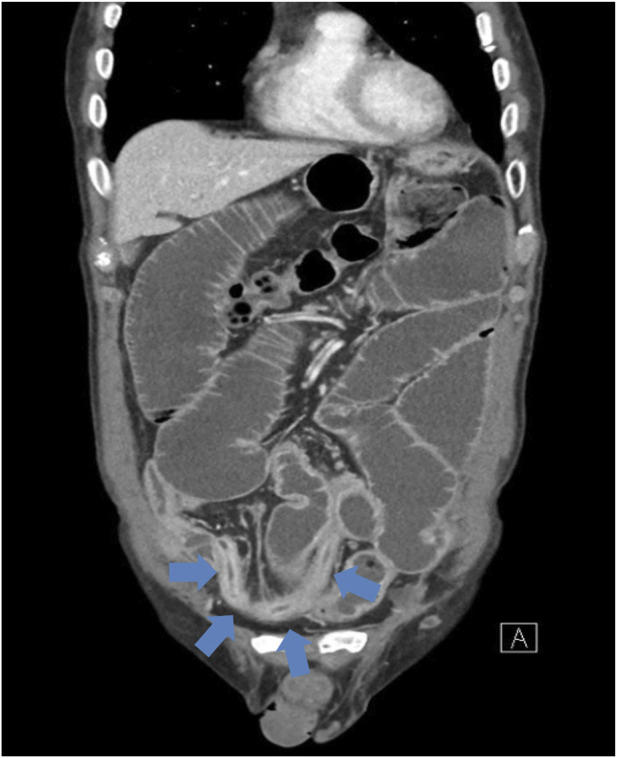
Coronal contrast-enhanced abdominal CT on HD 25 demonstrating multisegmental wall thickening and luminal narrowing from the distal jejunum to the distal ileum (arrows), compatible with inflammatory stricture and mechanical obstruction.

- Figure 1 Initial X-ray and Follow up X-ray on HD 13.
- Figure 2 2nd Abdominal CT (HD 25): Demonstrated worsening multisegmental wall thickening of the distal jejunum to the distal ileum, accompanied by lumen dilation and stricture.

Given the patient’s deteriorating mechanical obstruction despite conservative management, surgical exploration was planned and performed on HD 27.

### Surgical intervention and outcome

Exploratory laparotomy revealed severe adhesion among the small bowel loops in the lower abdomen and pelvis. Crucially, the terminal ileum was affected by an extensive, continuous global stricture extending approximately 80 cm proximally from the ileocecal valve, with evidence of skip lesional strictures extending up to 150 cm. Tubular adhesion suggestive of a fistula between the ileum and cecum was observed. The surgical team performed an ileocecectomy, resecting the severely damaged 132 cm segment of the small and large intestine, followed by an ileocecectomy. The small bowel remnant length was documented as approximately 200 cm.

**FIGURE 3:**
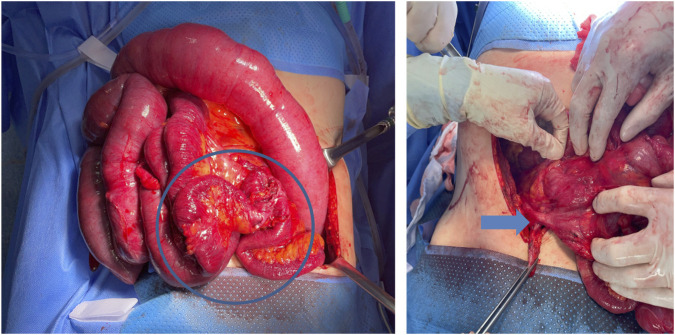
Intraoperative findings on HD 27 showing severe adhesions and a continuous global stricture of the terminal ileum extending proximally (left), with a suspected ileocecal fistula (right, arrow).

- Figure 3 OP findings (HD 27): continuous global stricture extending approximately 80 cm proximally from the ileocecal valve and fistula between the ileum and cecum.

Pathological examination of the resected specimen confirmed chronic, transmural inflammation with ulceration and adhesion in the ileum and cecum, consistent with severe chemical injury leading to irreversible fibrosis and stricture formation.

**FIGURE 4:**
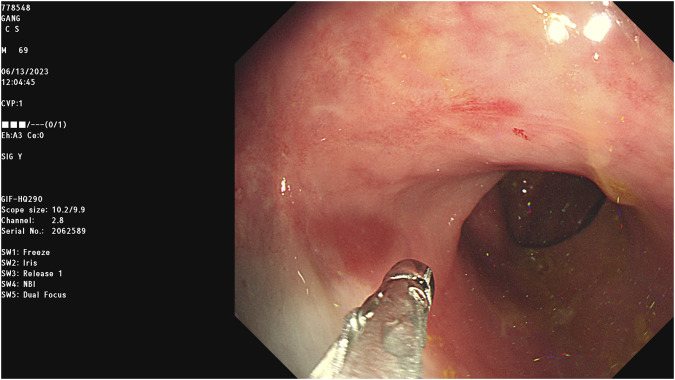
Sigmoidoscopy on the day of discharge demonstrating chronic inflammatory changes with erythema and erosion in the rectum; biopsy revealed chronic active inflammation.

On the day of discharge (13 June 2023), a follow-up sigmoidoscopy was performed for evaluation of the distal GI tract, revealing some scar change in the rectum. Biopsy findings confirmed the ongoing inflammatory process:

- Figure 4 Sigmoidoscopy (Discharge Day): Rectum (13 cm from anal verge) Chronic active inflammation with erosion and foveola epithelial hyperplasia, alongside hyperplastic foveola epithelium (suggestive of a hyperplastic polyp) and a spindle cell nodule in the lamina propria.

Post-operatively, the patient’s recovery was steady. The L-tube was removed on POD 2, and the patient was discharged on POD 14 (HD 42).

## Discussion

This case represents an extraordinary clinical manifestation of paint thinner intoxication, distinct from the typical presentation of acute, inhalation-induced systemic toxicity (Mickiewicz and Gomez, 2001; Agin et al., 2016). The patient’s course highlights two critical toxicological and surgical considerations.

First, the immediate sequelae of massive ingestion involved not only acute central nervous system (CNS) depression and hemodynamic instability but also direct chemical damage to the GI tract, evidenced by significant hematochezia and the presence of thinner-smelling stool.

Second, and most uniquely, the progression of the injury over 4 weeks to localized, severe, irreversible structural failure—stricture and fistula formation in the terminal ileum—mirrors the pathology typically associated with corrosive (acid or base) ingestion, not neutral volatile hydrocarbons. This unusual localization of damage (terminal ileum) compared to the common sites for corrosive injury (esophagus, stomach, duodenum) suggests a specific mechanism: the protective pyloric spasm, which normally limits the passage of toxic materials into the small intestine, was likely overcome. This loss of protective reflex was likely due to the massive volume ingested (500 mL) coupled with profound CNS depression from the co-ingestion of a large amount of alcohol. The toxic material (aromatic hydrocarbons) then traversed the upper GI tract and accumulated at the ileocecal valve, an area where content naturally stagnates, maximizing the contact time between the lipophilic solvents and the bowel wall. This prolonged contact led to transmural chemical necrosis, followed by chronic inflammation and subsequent pathological fibrosis and stricture.

The distinctive clinical message of this report pertains to management strategy. The patient stabilized systemically within 3 days but developed progressive mechanical obstruction over the next 24 days. In patients presenting with persistent or progressive mechanical sequelae, such as refractory recurrent ileus and hematochezia, following massive paint thinner ingestion, a high index of suspicion for developing chronic structural lesions (stricture/fistula) is warranted. This suggests that timely surgical intervention, rather than prolonged conservative management, may be critical to expedite patient recovery. The failure of extended conservative management in this case underscores the necessity of aggressive surveillance and intervention for advancing mechanical symptoms in this rare toxicological setting.

## Conclusion

This case report documents a rare instance of severe, delayed gastrointestinal structural complications—extensive stricture and fistula formation in the terminal ileum—following massive oral paint thinner ingestion in an adult. The severe, localized pathology necessitated an extensive ileocecectomy. This outcome serves as a critical reminder that while acute systemic signs may resolve, persistent mechanical symptoms like refractory ileus and hematochezia should trigger aggressive diagnostic workup (e.g., repeat CT) and consideration for early surgical intervention to prevent irreversible structural failure and minimize patient morbidity. Further reporting of such unique toxicological events is essential to refine management guidelines.

## Data Availability

The original contributions presented in the study are included in the article/supplementary material, further inquiries can be directed to the corresponding author.

## References

[B1] Agin K. Hassanian-Moghaddam H. Shadnia S. Rahimi H. R. (2016). Characteristic manifestations of acute paint thinner-intoxicated children. Environ. Toxicol. Pharmacol. 45, 15–19. 10.1016/j.etap.2016.05.001 27235798

[B2] Argo A. Bongiorno D. Bonifacio A. Pernice V. Liotta R. Indelicato S. (2010). A fatal case of a paint thinner ingestion: comparison between toxicological and histological findings. Am. J. Forensic Med. Pathol. 31 (2), 186–191. 10.1097/PAF.0b013e3181c6c11f 20010286

[B3] Beamon R. F. Siegel C. J. Landers G. Green V. (1976). Hydrocarbon ingestion in children: a six-year retrospective study. Jacep. 5 (10), 771–775. 10.1016/s0361-1124(76)80307-3 1018351

[B4] Dhibar D. P. Sahu K. K. Jain S. Kumari S. Varma S. C. (2018). Methemoglobinemia in a case of paint thinner intoxication, treated successfully with vitamin C. J. Emerg. Med. 54 (2), 221–224. 10.1016/j.jemermed.2017.10.035 29258784

[B5] Malingre M. M. Hendrix E. A. Schellens J. H. Koks C. H. Tibben M. M. Challa E. E. (2002). Acute poisoning after oral intake of a toluene-containing paint thinner. Eur. J. Pediatr. 161 (6), 354–355. 10.1007/s00431-001-0889-1 12029459

[B6] Mickiewicz M. Gomez H. F. (2001). Hydrocarbon toxicity: general review and management guidelines. Air Med. J. 20 (3), 8–11. 11331818

[B7] Rahimi H. R. Agin K. Shadnia S. Hassanian-Moghaddam H. Oghazian M. B. (2015). Clinical and biochemical analysis of acute paint thinner intoxication in adults: a retrospective descriptive study. Toxicol. Mech. Methods 25 (1), 42–47. 10.3109/15376516.2014.975388 25297831

[B8] Tormoehlen L. M. Tekulve K. J. Nanagas K. A. (2014). Hydrocarbon toxicity: a review. Clin. Toxicol. (Phila) 52 (5), 479–489. 10.3109/15563650.2014.923904 24911841

[B9] Truemper E. Reyes de la Rocha S. Atkinson S. D. (1987). Clinical characteristics, pathophysiology, and management of hydrocarbon ingestion: case report and review of the literature. Pediatr. Emerg. Care 3 (3), 187–193. 10.1097/00006565-198709000-00015 3313305

[B10] Zaidi S. A. Shaw A. N. Patel M. N. Shah V. V. Rajendran D. Shah B. P. (2007). Multi-organ toxicity and death following acute unintentional inhalation of paint thinner fumes. Clin. Toxicol. (Phila) 45 (3), 287–289. 10.1080/15563650601031791 17453883

